# Infectivity of Chronic Malaria Infections and Its Consequences for Control and Elimination

**DOI:** 10.1093/cid/ciy055

**Published:** 2018-05-10

**Authors:** Ricardo Aguas, Richard J Maude, M Gabriela M Gomes, Lisa J White, Nicholas J White, Arjen M Dondorp

**Affiliations:** 1 Centre for Tropical Medicine and Global Health, Nuffield Department of Medicine, University of Oxford, United Kingdom; 2 Mahidol-Oxford Tropical Medicine Research Unit, Faculty of Tropical Medicine, Mahidol University, Bangkok, Thailand; 3 Harvard T.H. Chan School of Public Health, Harvard University, Boston, Massachusetts; 4 Liverpool School of Tropical Medicine, United Kingdom; 5 CIBIO-InBIO, Centro de Investigação em Biodiversidade e Recursos Genéticos, Universidade do Porto, Portugal

**Keywords:** relative infectivity, chronic infections, *Plasmodium falciparum*, malaria elimination

## Abstract

Assessing the importance of targeting the chronic *Plasmodium falciparum* malaria reservoir is pivotal as the world moves toward malaria eradication. Through the lens of a mathematical model, we show how, for a given malaria prevalence, the relative infectivity of chronic individuals determines what intervention tools are predicted be the most effective. Crucially, in a large part of the parameter space where elimination is theoretically possible, it can be achieved solely through improved case management. However, there are a significant number of settings where malaria elimination requires not only good vector control but also a mass drug administration campaign. Quantifying the relative infectiousness of chronic malaria across a range of epidemiological settings would provide essential information for the design of effective malaria elimination strategies. Given the difficulties obtaining this information, we also provide a set of epidemiological metrics that can be used to guide policy in the absence of such data.

## Teaser

The relative infectiousness of chronic malaria infections determines the likelihood of success of different malaria elimination strategies.

In human feeding experiments, the measured infectivity of patent infections to mosquitoes seems to fluctuate significantly over time and not be linearly correlated with peripheral blood gametocyte counts [1]. In fact, several mosquito blood-feeding assays have shown that low gametocyte counts in humans do not preclude infection of mosquitoes, with a significant proportion of submicroscopic gametocytemias resulting in successful oocyst development in mosquitoes [2–6]. The data available suggest a sigmoidal relationship between human infectivity to mosquitoes and gametocyte density, with significant variation in infectivity levels at low gametocyte densities [7, 8]. Several estimates of the contribution of these submicroscopic gametocyte carriers to overall transmission reinforce that these infections are, in fact, nonnegligible [4, 5, 9, 10]. An analytical study that examined these infectivity trials has found that for low levels of transmission, submicroscopic infections could potentially be the source of 20%–50% of all human-to-mosquito transmissions [11]. 

Another study shows that gametocyte density can be larger in asymptomatic individuals than in some symptomatic patients [12]. That study also reported increased infectivity of asymptomatic gametocyte carriers not correlated with gametocyte densities, suggesting that other factors such as gametocyte maturity and/or human blood factors might influence their infectivity [13–15]. This might help explain why high gametocyte densities do not necessarily result in mosquito infections [2, 6, 10]. One recent analysis of longitudinal data from Dielmo, Senegal, showed that gametocyte densities increased as malaria prevalence decreased from 70% to 20% [16], further highlighting how factors determining the sexual commitment of the malaria parasite can confound the assumptions of how asexual parasitemia translates into human infectivity to mosquitoes [17]. In summary, there is no consensus on the relative infectivity of chronic malaria infections compared with clinical infections, owing to sparseness of data, difficulties in measuring individuals’ infectivity over time, and the variable relationship between asexual parasitemia, gametocytemia, and infectivity.

## EFFECT OF HETEROGENEOUS INFECTIVITY ON MALARIA TRANSMISSION

Accepting that clinical malaria infections are, on average, considerably shorter in duration owing to treatment, and that chronic infections can extend up to several months, it is evident that, if clinical and chronic infections had similar infectivity, chronic infections would be the main contributors to overall malaria transmission. This has been explored theoretically in the past [18] and has helped in determining the limits for malaria control in low-transmission areas. That study presented a mathematical malaria transmission model that was used to fit age profiles of clinical malaria in 8 sub-Saharan Africa (SSA) regions of varying endemicity, to make inferences on key transmission parameters, such as region-specific entomological inoculation rates (EIRs), as well as setting independent parameters, such as the mean duration of clinical and chronic infections. 

This allowed the extrapolation of the relative infectiousness of chronic malaria infections, given specific relative infectivity (*ϕ*) values. The estimated parameters suggested that chronic infections were 6 times longer in duration than clinical ones. Assuming *ϕ* to be 1, infectiousness would be 6 times higher in chronic infections. This means that in a clinically immune population, a single index case would generate 6 times more secondary infections than in a completely susceptible population, because the majority of infections would be chronic in the former and clinical in the latter. This difference translates into a phenomenon termed *bistability,* characterized by the existence of 2 stable equilibria for a given parameter set.

Published estimates of infectiousness period length were derived from African data sets, with chronic infections thought to last about 150 days on average [18–20]. Although these estimates have accounted for differences in exposure across populations in Africa, they are not necessarily applicable to the Greater Mekong Subregion (GMS). Preliminary unpublished data exploring the duration of chronic carriage in a low-transmission setting in Vietnam suggest that infections in the GMS are slightly shorter in duration (approximately 100 days). This difference could be driven by disparities in parasite population structures. Parasite genetic diversity has been described as lower in Southeast Asia than in African settings with the same transmission intensity [21]. Although this fits with the suggested lower gene flow in Southeast Asia [22, 23], genetic factors are only a single determinant among many that could account for differences in the period of infectiousness across malaria-endemic areas. 

Another well-documented significant difference between SSA and the GMS is their markedly distinct vector bionomics. Whereas the most abundant vectors in Africa are endophilic and antropophilic, in Southeast Asia vectors most commonly bite outdoors and do not preferentially bite humans [24–26]. This has major implications for the effectiveness of any vector-based control strategy, specifically insecticide-treated bed nets (ITNs) [27, 28] and indoor residual spraying (IRS).

**Figure 1.**
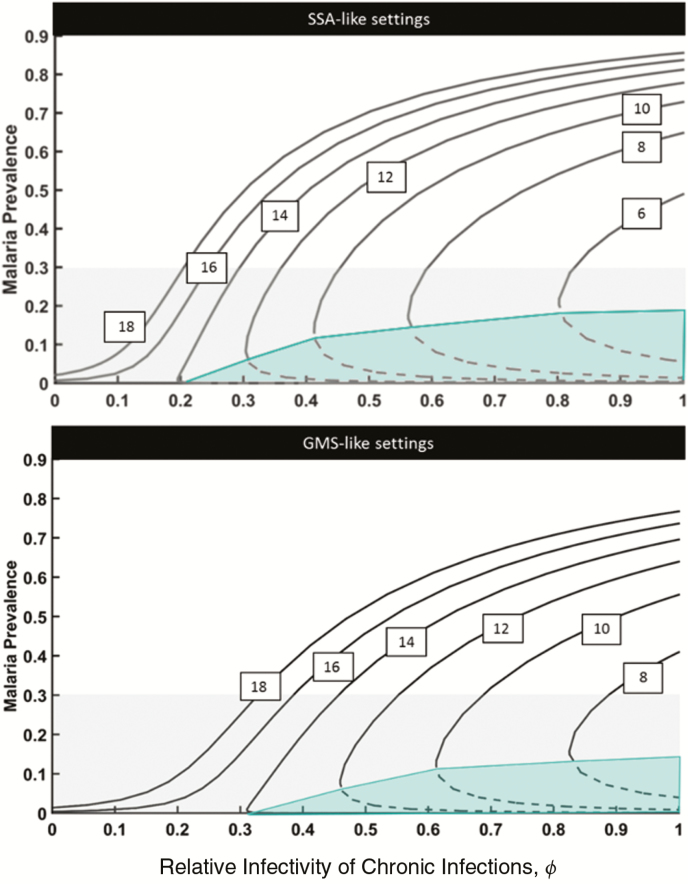
Malaria equilibrium prevalence for different combinations of vectorial capacity (VC) and relative infectivity of chronic infections. Curves from left to right represent decreasing values of VC (18, 16, 14, 12, 10, 8, and 6 infectious bites per year) as indicated in the text boxes. Dashed lines reflect unstable equilibrium solutions (ie, elimination thresholds; solid lines, stable equilibrium solutions). The grey area illustrates the lower end of the malaria transmission spectrum which is usually deemed “controllable.” The green area delimits areas of unstable malaria transmission where malaria is expected to be eliminated over time without additional control measures. Abbreviations: GMS, Greater Mekong Subregion; SSA, sub-Saharan Africa.

In this article we use a previously developed theoretical framework [18] to explore the predicted malaria prevalence at equilibrium across a range of relative infectivity values (*ϕ*) and vectorial capacities (VCs) (Figure 1). We do so for 2 distinct settings meant to characterize the generic features of malaria transmission in SSA and GMS settings. The 2 prototypical settings differ only in the assumed length of chronic infectiousness and vector control effectiveness, with SSA-like settings having an infectiousness period of 165 days versus 100 days for GMS-like settings. As mentioned above, vector control is much more “leaky” in the GMS, translating into an implemented 25% reduction in infection risk for the modeled vector control packages in the GMS, compared with an 80% reduction for the SSA simulations.

The model shows, as expected, that for a fixed value of *ϕ* malaria prevalence is predicted to increase as VC increases. Of more relevance, however, is that low levels of endemicity (ie, <30% prevalence) could occur as a result of a very large number of combinations of both VC and *ϕ* values. This means that a 20% malaria prevalence, for example, can result from a very large VC with the assumption that chronic infections are virtually noninfectious, much lower VC with chronic infections being the main infection reservoir, or a range of intermediate scenarios. Details on all used model variables and key definitions mentioned throughout the article can be found in Table 1.

**Table 1.** Key Definitions

Term	Definition
VC	Vectorial capacity (the average number of infectious bites arising from mosquitoes originally infected by a single infectious person per day), formally defined as follows: $VC=\frac{ma^{2}e^{-gn}}{g},$ where *m* is the number of mosquitoes per person; *a,* daily mosquito biting rate; and *n,* duration of the mosquito sporogony cycle; and *g,* mosquito life expectancy in days
EIR	Entomological inoculation rate (average number of infectious mosquito bites per person per year), formally defined as follows: EIR=VC(cp+cϕ(1−p)), where *p* is the proportion of infections that result in clinical malaria; and *c,* per-bite probability of infectious individuals with clinical manifestations to infect mosquitoes (commonly referred to as infectivity of humans to mosquitoes).
ϕ	Relative infectivity of chronic compared with clinical infections
Infectiousness	Overall transmissibility of 1 malaria infection over the course of the time when the infected person is infective to mosquitoes
*R*_0_	Basic reproduction number, formally defined as follows: $R_{0}=VCb\left[\frac{cp}{D_{c}}+\frac{c\phi (1-p)}{D_{a}}\right],$ where *b* is the per-bite probability that an infectious mosquito infects a susceptible human; *D*_*c*_, mean duration of infectiousness for clinical infections; and *D*_*a*_, mean duration of infectiousness in asymptomatic individuals
ContrA	Contribution of chronic infections to overall malaria transmission; defined as follows: $\frac{(1-p)\phi D_{a}^{-1}}{pD_{c}^{-1}+(1-p)\phi D_{a}^{-1}}$
AgeR	Ratio between malaria prevalence in children aged <10 y and all-age malaria prevalence
ITN	Insecticide-treated bed net
IRS	Indoor residual spraying
MDA	Mass drug administration in the form of 3 rounds of artemisinin combination therapy treatment
Bistability	Characterized by the existence of 2 stable equilibria for a given parameter set; the dynamics of the system converge to either the disease-free or the endemic equilibrium depending on the initial conditions (namely, initial prevalence); if the endemic state is perturbed past the unstable equilibrium solution (eg, through MDA), the system can converge to the disease-free equilibrium over time

## USE OF OTHER EPIDEMIOLOGICAL METRICS TO DETERMINE THE CONTRIBUTION OF CHRONIC INFECTIONS TO MALARIA TRANSMISSION

The relative infectivity of chronic malaria infections remains largely unknown. There is a pressing need for high-quality data that can inform such a crucial driver of malaria transmission, which will require longitudinal follow-up studies in both clinical and chronic infections, with serial mosquito-feeding assays. As we approach the elimination targets set by some GMS countries (as early as 2020), we need to make use of other more easily accessible data to advise national malaria control programs’ elimination strategies. Supplementary Figure S1 shows how different sets of VC and *ϕ* pairs lead to different predicted values for EIR, proportion of clinical malaria cases treated, all-age prevalence, percentage contribution of chronic infections to overall transmission, and the ratio of malaria prevalence in children <10 years old to all-age malaria prevalence. These data (or at least some of them) are routinely collected and could circumvent the gap in our knowledge of what value *ϕ* should take. If we know the EIR, the proportion of clinical malaria cases treated, and the ratio of malaria prevalence in children <10 years old to all-age malaria prevalence in any specific setting, we can easily place that setting on our VC-*ϕ* coordinate system.

## OPTIMAL CONTROL STRATEGIES FOR A GIVEN TRANSMISSION INTENSITY

The prospects for malaria control has been thoroughly explored analytically elsewhere [18], and its predictions have since been revisited [29]. In essence, assuming *ϕ* to be 1, there is a range of VC values for which stable elimination can be reached solely through the administration of effective antimalarial drugs to asymptomatic individuals, without the need for any additional vector control efforts. This is illustrated by the green-shaded areas in Figure 1. The dashed black line represents the elimination threshold, that is, the malaria prevalence that must be achieved through treatment of both asymptomatic and symptomatic individuals. after which control measures can be relaxed and elimination is still achieved over time. If chronic infections are not transmissible (φ=0), however, treatment of asymptomatic parasite carriers is completely ineffective, and vector control strategies (and potentially vaccination) emerge as the only means to halt transmission (left-most curves in Figure 1).

**Figure 2.**
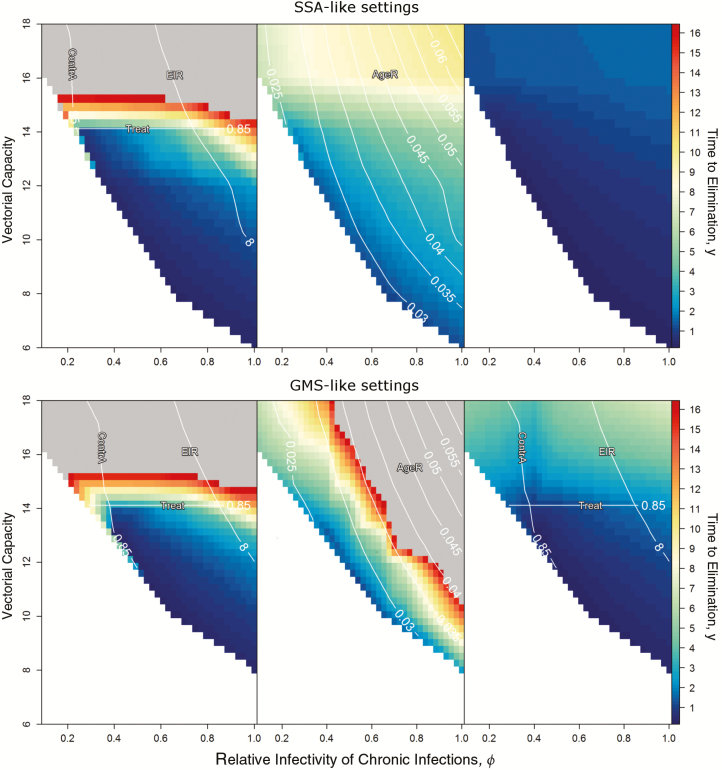
Surfaces illustrating the minimum time required for elimination to be reached for a given combination of vectorial capacity and relative infectivity (*ϕ*). Colors reflect the time to elimination in years (the minimum time a given intervention must be sustained for elimination to be reached); this does not reflect the time it takes for malaria to be eliminated, because an intervention could be required to be sustained for only 1 year, and then relaxed, for example, with elimination following after another 3 months. The contour lines indicate epidemiological metrics useful to determine the feasibility of control strategies in the simulated settings. Abbreviations: AgeR, ratio between malaria prevalence in children aged <10 y and all-age malaria prevalence; ContrA, contribution of chronic infections to overall malaria transmission; EIR, entomological inoculation rate; GMS, Greater Mekong Subregion; SSA, sub-Saharan Africa; Treat, proportion of clinical malaria cases treated.

Figure 2 shows the timelines for elimination by deploying mass drug administration (MDA), vector control or MDA plus vector control strategies, assuming different combinations of VC and *ϕ* for the 2 explored settings. These MDA elimination strategies consist of annual MDA campaigns of 3 monthly rounds each, whereas vector control strategies consist of integrated packages, including annual ITN distribution and IRS deployment campaigns that reduce the VC by 80% in SSA-like settings and by 25% in GMS-like settings. It is clear that integrated control programs with both MDA and vector control components, are predicted to be by far the most efficient in reducing transmission to the point of elimination in a reasonable time frame for any parameter set. Although drug-centric approaches aimed at reducing the pool of chronic parasitemia can be the most effective strategy for a wide range of VC-*ϕ* combinations, for parameter combinations of high VC and low *ϕ*, the bistability region is lost, and elimination is rendered impossible with drug-centric approaches only. As expected, vector control–centric approaches have much less impact in GMS-like setting simulations and are predicted to be insufficient to eliminate malaria by themselves for high values of both VC and *ϕ*. Broadly, a few patterns emerge for potential operational programs considering different elimination horizons.

### <5 Years

In SSA settings, control programs could choose either MDA or vector control for the lower end of EIRs to meet their elimination goal, because there is no combination of VC and *ϕ* values for which MDA outperforms vector control. For GMS-like settings, stand-alone vector control approaches are viable only for very low EIRs, but vector control is still needed in combination with MDA to enable malaria elimination at high VC values.

### ≥5 Years

**Figure 3.**
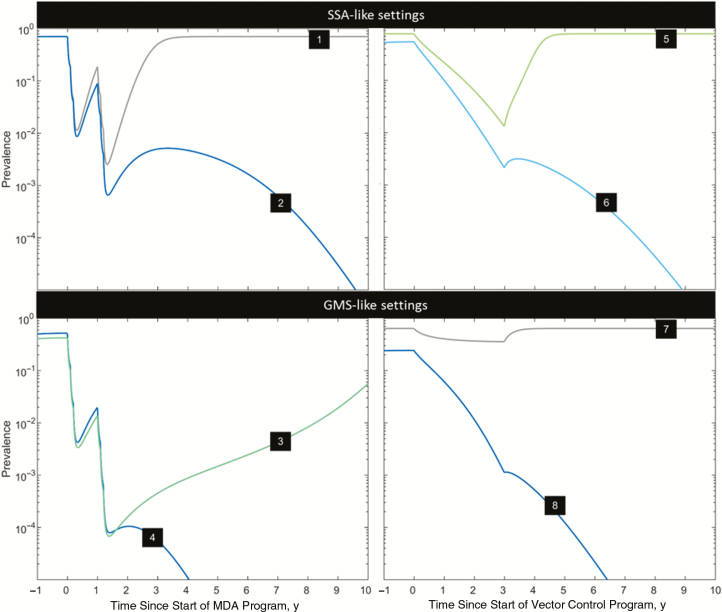
Time dynamics of interventions for the combinations of vectorial capacity and relative infectivity highlighted 1–8 in Figure 2. The panels on the left illustrate 2 MDA campaigns (3 rounds each) in consecutive years; those on the right, annual insecticide-treated bed net and indoor residual spraying campaigns over a period of 3 years. Colors reflect those of the contour plots in Figure 2, indicating the predicted time to elimination. Abbreviations: GMS, Greater Mekong Subregion; MDA, mass drug administration; SSA, sub-Saharan Africa.

Combined control strategies are only required for highest values of VC and *ϕ* in any setting. In GMS-like settings, MDA outperforms vector control for combinations of low VC and high *ϕ*. Sustaining MDA campaigns for more than a couple of years is not realistic owing to logistical considerations and concerns about the emergence or spread of drug resistance. We have thus focused on the potential impact of elimination strategies consisting of 2 annual MDA campaigns of 3 artemisinin combination therapy (ACT) rounds each and vector control intervention packages consisting of annual ITN and IRS campaigns (Figure 3). 

We chose parameters sets that illustrate the 3 possible outcomes of elimination driven intervention strategies: successful elimination (lines 2 and 4) with the 2 annual MDA campaigns or 3 years of sustained vector control (note that elimination might only occur a few years after intervention has been halted); failure to eliminate where elimination is theoretically possible (lines 3 and 5) because the intervention effort was halted too soon, with resurgence following after a few years; and elimination impossible (lines 1 and 7), with malaria returning to baseline prevalence levels soon after the intervention stops. 

One important factor to consider here is the speed at which MDA and vector control strategies reduce malaria prevalence. By definition, MDA campaigns consist of administering antimalarial drugs to as many individuals as possible, regardless of their infection status, producing a dramatic effect on malaria prevalence over a short period of time. If elimination is not achieved in the 12 months after MDA, then it is very unlikely to ever happen. Vector control, on the other hand, has a more diluted impact on transmission by affecting the likelihood that existing infections generate other infections. However, it has the advantage of being able to decrease the basic reproduction number (*R*_0_) past the *R*_0_ = 1 threshold, thus bringing the system to the bistability region for VC values >14 and potentially making elimination viable for a wider spectrum of VC values.

By overlaying the epidemiological metrics displayed in Supplementary Figure S1 onto the elimination surfaces in Figure 2, we get very convenient measures of elimination feasibility that can guide national malaria control programs in the absence of reliable data on the relative infectivity of chronic infections. These are shown as white lines in Figure 2 and help delimit the parameter space in which elimination can occur within a reasonable time frame (blue-colored surface).

For MDA approaches, elimination can be achieved with 2 annual MDA campaigns if (1) baseline clinical case management is very good, with 85% of all clinical cases getting treatment; (2) EIR is <8; and (3) chronic infections are the main contributors to malaria transmission, with 85% of all infections originating in asymptomatically infected individuals. Strikingly, this is consistent in both SSA-like and GMS-like settings. For vector control approaches, elimination can be achieved with a 3 year blanket intervention package of ITN and IRS if (1) baseline clinical case management is very good, with 85% of all clinical cases getting treatment, and (2) children <10 years old sustain ≤3.5% of all infections for SSA-like settings, or ≤2.6% for GMS-like settings. Of the tipping points presented here, the baseline clinical management coverage emerges as the most promising parameter for national control programs to adjust in order to increase their likelihood of success. 

Supplementary Figure S2 shows how elimination can become feasible for higher VC settings by increasing the baseline treatment rates. It reflects the minimum treatment rate one would need to implement 1 year before an elimination strategy consisting of 2 annual MDAs, for elimination to be achieved. This highlights how good malaria case management should be the foundation of any malaria control program. If we consider areas where case management rates are poor (<50% of cases receiving treatment), with little to no vector control, we can explore 3 hierarchical intervention strategies: (1) increasing case management rates to 85%, (2) strategy 1 plus implementation of vector control packages decreasing VC by 80% in SSA and 25% in the GMS, and (3) strategy 2 plus deployment of 1 MDA campaign (3 ACT rounds).

**Figure 4.**
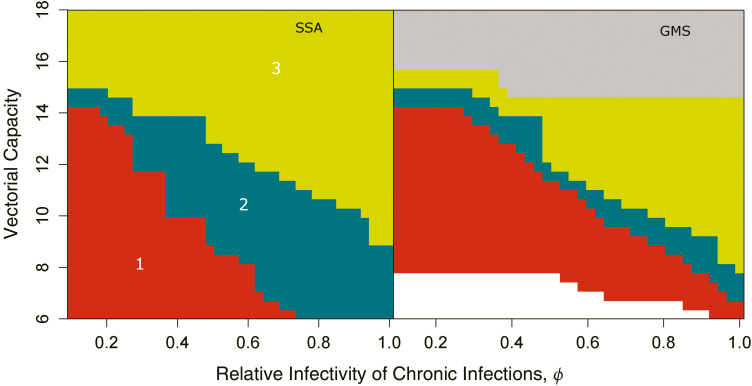
Minimum strategy required for elimination to be reached. Strategies are hierarchical, starting with improved case management (1-red), and ultimately also including vector control (2-teal) and mass drug administration campaigns (3-green). Gray represents parameter sets for which no intervention package is successful in achieving elimination. Abbreviations: GMS, Greater Mekong Subregion; SSA, sub-Saharan Africa.

Model predictions indicate that improved case management alone (strategy 1) could lead to elimination in a significant range of VC-*ϕ* parameter combinations (Figure 4). In fact, elimination can be achieved without the need for any MDA campaign in about half the settings where malaria is deemed “controllable.” The lower efficiency imposed on simulated vector control strategies for the GMS is evidenced by how narrow region 2 in Figure 4 is, but also by the large gray area, depicting the parameter region where even strategy 3 is predicted to be insufficient to reach elimination. Novel vector control tools that circumvent efficiency gaps in currently existing tools could then potentially expand both areas 2 and 3 in the right panel in Figure 4.

## DISCLAIMER

Here we provide a qualitative analysis of how the predicted success of elimination strategies in SSA or GMS settings is extremely sensitive to a biological parameters for which we have no rigorous estimate. We emphasize that the quantitative aspects of these simulations do not necessarily reflect what would be seen in the field due to region-specific factors, such as spatial transmission heterogeneity, individual infection risk heterogeneity, and intervention logistical constraints (none of which are taken into account here). The significant tradeoff between complexity and tractability renders it impossible to analytically solve sophisticated models that take into account vector and human population dynamics with integrated within host dynamics. 

The simple model presented here has several limitations, mainly with respect to how it fails to capture heterogeneity. The main source of heterogeneity lacking in the simple model pertains to the transmission network describing the human-mosquito interface. Infection risk is known to be heterogeneous across individuals and is likely to be clustered (depending on mosquito habitat dispersal). These factors are well known to have an impact on dynamical systems’ behavior, but their impact on the likelihood and timeline of elimination is complex. The VC numbers presented throughout the article can be translated into a mean risk of infection (which is the same for each individual in the simple model explored here). 

If we consider a realistic scenario, wherein some individuals are at greater risk and others can be at no risk, keeping the same mean risk of infection, we decrease the likelihood of reaching elimination, because transmission can be sustained by the individuals with a greater than mean risk of infection. Supplementary Figure S3 shows the results of a very sophisticated individual-based model that assumes a skewed log-normal distribution of risk, wherein the majority of individuals are at little to no risk but some are constantly receiving infections, thus providing a more realistic but simultaneously “worst case” scenario. In this extreme example, the elimination parameter space is quite contracted, but the relative space attributable to each strategy is maintained. In this article, we intend not to make direct recommendations to national malaria control programs but rather to demonstrate how any rigorous recommendations considering detailed country-specific data are dependent on our assumptions about the duration of the infectious period and the relative infectivity of chronic infections.

## CONCLUSIONS

The relative infectivity of chronic infections has severe consequences for malaria elimination prospects. Reliable estimates to inform that parameter will require longitudinal follow-up studies of sufficient duration in both clinical and chronic infections, with serial mosquito-feeding assays. Although we appreciate the time-consuming efforts involved and the significant logistical burden (specifically on the insectary) required to perform such a study, we consider those concerns to be greatly outweighed by the dramatic expected impact on malaria elimination policy. In the absence of such data, the presented model offers guidance as to what other more easily measurable epidemiological data might best inform elimination strategies’ likelihood of success. We also highlight how good case management should be the foundation of any malaria control strategy, having the potential to lead to elimination by itself in some settings.

## Supplementary Data

Supplementary materials are available at *Clinical Infectious Diseases* online. Consisting of data provided by the authors to benefit the reader, the posted materials are not copyedited and are the sole responsibility of the authors, so questions or comments should be addressed to the corresponding author.

Supplementary Figure 1

Supplementary Figure 2

Supplementary Figure 3

Supplementary Data

## Notes

***Disclaimer***. The funding sources did not influence the contents of the manuscript in any way.

***Financial support.*** This work was supported by the Bill and Melinda Gates Foundation (gatesfoundation.org/) (grant BMGF OPP1110500) and was part of the Wellcome Trust–Mahidol University–Oxford Tropical Medicine Research Programme, funded by the Wellcome Trust of Great Britain (wellcome.ac.uk/) (grant 101148/Z/13/Z).

***Potential conflicts of interest.*** All authors: no reported conflicts of interest. All authors have submitted the ICMJE Form for Disclosure of Potential Conflicts of Interest. Conflicts that the editors consider relevant to the content of the manuscript have been disclosed.
