# Supplementary figures and images for: Infectivity of Chronic Malaria Infections and Its Consequences for Control and Elimination
Source: Clin Infect Dis. 2018 May 10;67(2):295–302. doi: 10.1093/cid/ciy055 (PMC6030896; doi:10.1093/cid/ciy055)

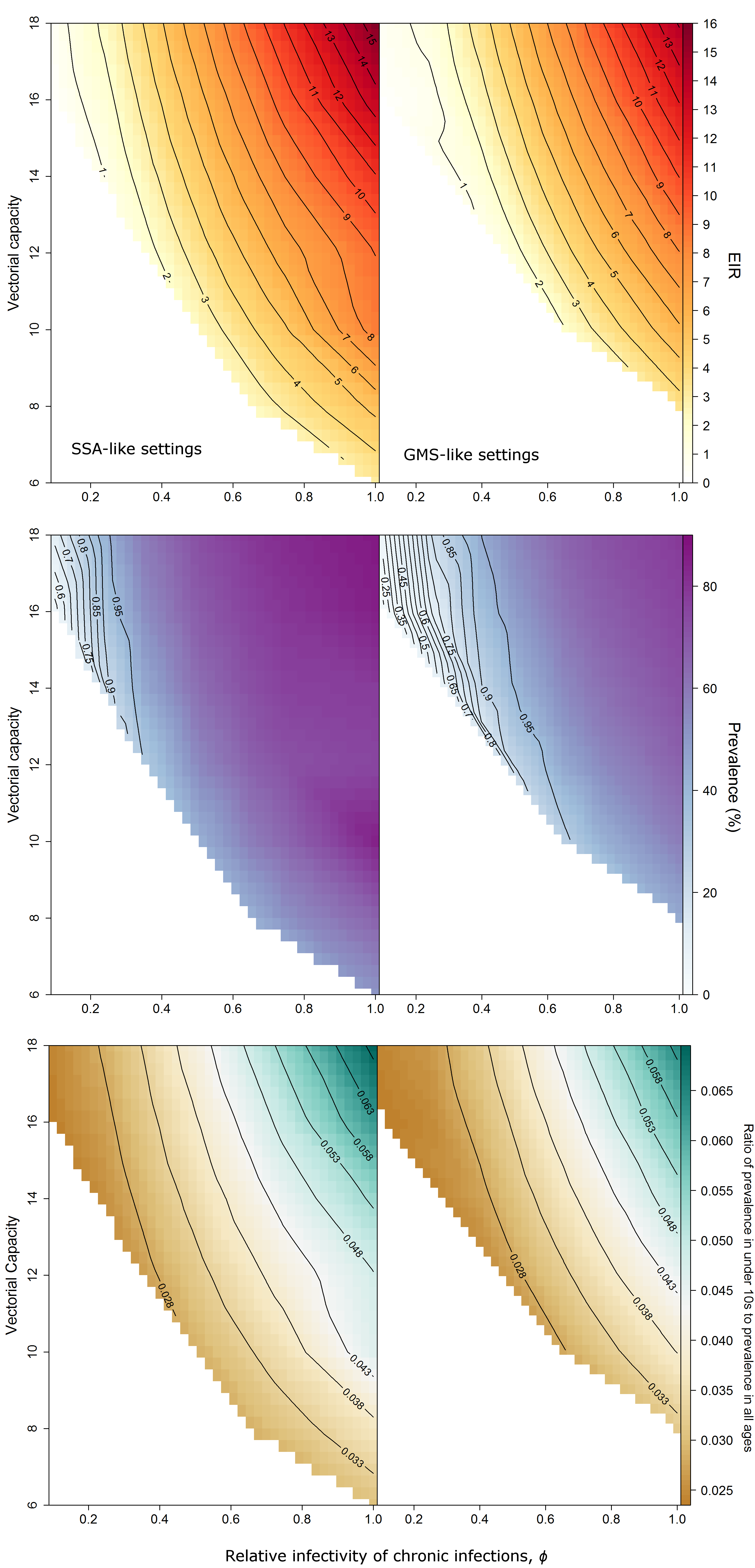

Supplement: Supplementary Figure 1 [file ciy055_suppl_supplementary_figure-1.png]

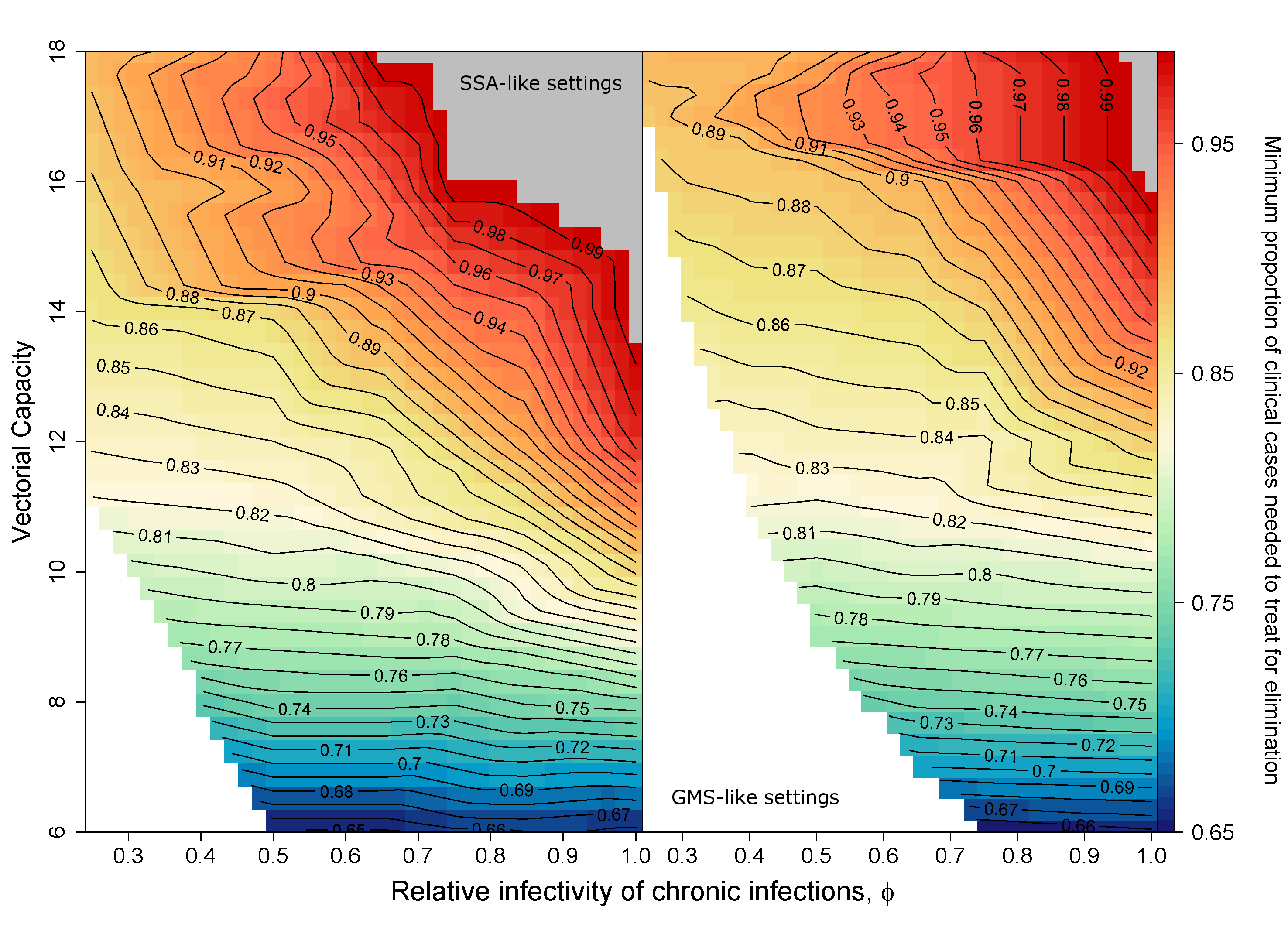

Supplement: Supplementary Figure 2 [file ciy055_suppl_supplementary_figure-2.png]

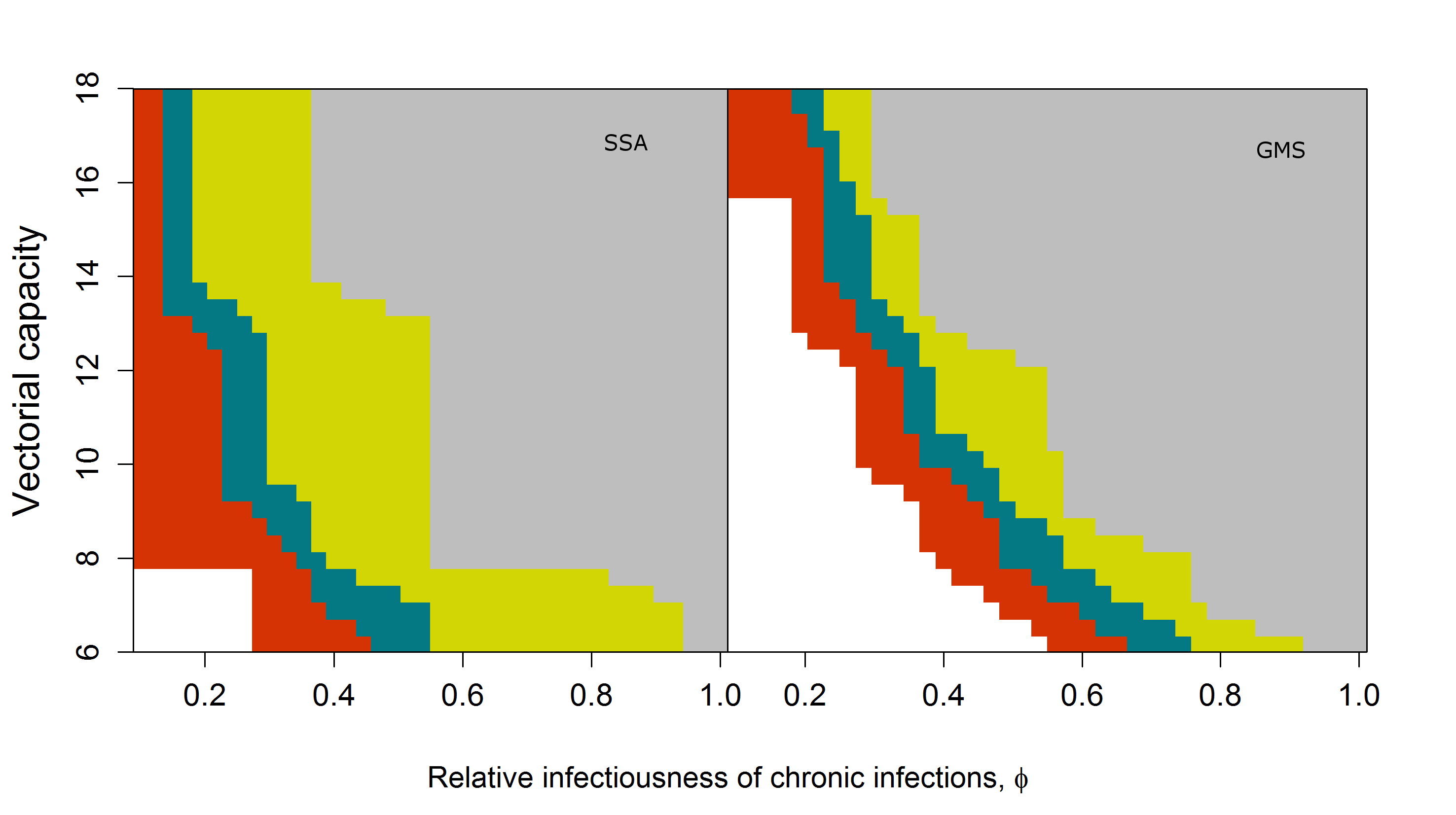

Supplement: Supplementary Figure 3 [file ciy055_suppl_supplementary_figure-3.png]
