# Supplementary material for: Infectivity of Chronic Malaria Infections and Its Consequences for Control and Elimination
Source: Clin Infect Dis. 2018 May 10;67(2):295–302. doi: 10.1093/cid/ciy055 (PMC6030896; doi:10.1093/cid/ciy055)
Supplement: Supplementary Data [file ciy055_suppl_supplementary_data.docx]

Supplementary Data

Supplementary Figure 1. Epidemiological metrics that emerge as a result of specific VC, *φ* combinations. The top row shows the model predicted EIR for each VC and *φ* set. The middle panels illustrate the resulting all-age malaria prevalence and show contours (black lines) for the contribution of chronic infections to transmission (*ContrA*). The bottom row displays the ratio between malaria prevalence in children under the age of 10 and all-age malaria prevalence (*AgeR*).

Supplementary Figure 2. Minimum baseline case management effort required for elimination to be possible with two MDA campaigns (3 rounds each) in consecutive years.

Supplementary Figure 3. Minimum strategy required for elimination to be reached using an individual based, discrete time, spatially explicit, stochastic model, with mosquito population dynamics and human population movement. Strategies are hierarchical starting with improved case management and ultimately also including vector control and MDA campaigns. Grey indicates parameter sets for which no intervention package is successful in achieving elimination.
